# Impact of dissociation and interpersonal functioning on inpatient treatment for early sexually abused adults

**DOI:** 10.3402/ejpt.v4i0.22825

**Published:** 2013-12-30

**Authors:** Ellen K. K. Jepsen, Willie Langeland, Trond Heir

**Affiliations:** 1 Department for Trauma Treatment and Research Institute, Modum Bad, Vikersund, Norway; 2 Department of Psychiatry, Vrije University Medical Center, Amsterdam, The Netherlands; 3 Department of Psychiatry, University of Oslo, Oslo, Norway

**Keywords:** Early interpersonal trauma, inpatients, complex dissociative disorders, general psychiatric symptoms, interpersonal outcome

## Abstract

**Background:**

Little is known about the possible predictors of treatment outcome in early chronically sexually abused adults. The current study aimed to investigate what impact initial levels of dissociation and pre-treatment negative change in interpersonal functioning have on treatment response after 3 months of first-phase trauma inpatient treatment as well as after a period of 1 year the patients returned to their usual lives.

**Methods:**

The sample comprised 48 inpatients with childhood sexual abuse histories and mixed trauma-related disorders who were examined at discharge and prospectively followed up for a period of 1 year under naturalistic conditions. Outcome variables were general psychiatric symptoms and interpersonal problems as measured with the Symptom Check List-Revised (SCL-R) and the Inventory of Interpersonal Problems (IIP) Circumplex.

**Results:**

The central findings were that pathological dissociation and deterioration in interpersonal functioning prior to admittance predicted general psychiatric symptom levels and interpersonal problems at the end of treatment and at 1-year follow-up. Pathological dissociation, involving memory and identity problems, alone predicted negative outcome at the end of treatment. The findings at 1-year follow-up indicate that it is not pathological dissociation in isolation that affects outcomes, but rather the interaction between dissociation and change in interpersonal functioning prior to treatment.

**Conclusion:**

These findings indicate the need of addressing dissociation and interpersonal problems in treatment planning and favor an integrated treatment approach for complex trauma patients. Future research should investigate whether and how this leads to better outcome, including long-term maintenance of gains after the end of treatment.

Adult survivors of childhood sexual abuse (CSA) are likely to present with problems such as posttraumatic stress symptoms, depression, anxiety, somatization, suicidality, eating disorders, substance abuse, interpersonal dysfunction, revictimization, and self-mutilation (see Manglio, 2009, for a review). Furthermore, when primary caregivers or other trusted people were both the source of safety and attachment and the source of threat and violence, it may cause disorganized attachment and dissociation, involving relational problems (Alexander, 1992; Barach, 1991; Lyons-Ruth, Dutra, Schuder, & Bianchi, 2006). Adults with posttraumatic stress disorder (PTSD), dissociative disorders, and other symptomatology linked to chronic CSA, particularly those who were previously therapy resistant in outpatient settings, may seek inpatient treatment for their psychological problems.

Several outcome studies among adult inpatients with child abuse-related disorders demonstrate significant reduction of general psychiatric symptoms (e.g., Jepsen, Langeland, Sexton, & Heir, 2013; Jepsen, Svagaard, Thelle, McCullough, & Martinsen, 2009; Lampe & Gast, 2012; Stalker, Palmer, Wright, & Gebotys, 2005; Wright, Woo, Muller, Fernandes, & Kraftcheck, 2003) as well as significant improvement in interpersonal functioning (e.g., Jepsen et al., 2009, 2013). However, most of these studies also reported that a non-trivial number of patients did not respond to treatment (see Ali & Smartt, 2009, for a review). This individual variability in treatment response suggests the need to identify those patients with polysymptomatology related to child abuse who may need alternate treatment approaches to improve outcome. To increase our understanding of factors that may predict who will improve in treatment and maintain their gains after they return to their usual lives, the current study will focus on two possible predictors of treatment outcome in early chronically sexually abused adults with trauma-related disorders: dissociation and interpersonal problems (see Baars et al., 2011).

Findings have been inconsistent in determining whether dissociation interferes with the effectiveness of treatment for (complex) PTSD related to childhood abuse. Virtually, all of these studies have been based on outpatient samples. Some studies found that outpatients with severe levels of dissociation may need specific treatments (Cloitre, Petkova, Wang, & Lassell, 2012; Resick, Suvak, Johnides, Mitchell, & Iverson, 2012), whilst other studies (Dorrepaal et al., 2012; Hagenaars, Van Minnen, & Hoogduin, 2010) report no association between severity of dissociation and treatment effectiveness among outpatients. These studies used outcome measures for dissociation and PTSD symptoms. In addition, drawing from a sample of individuals with complex PTSD, Lynch, Forman, Mendelsohn, and Herman (2008)) found that the initial level of dissociation was not significantly associated with change in general psychiatric symptoms such as depression or self-harming behavior, during or after outpatient treatment. However, a study by Brand and Stadnik (2013) of dissociative disorder outpatients showed that initially high levels of dissociation did not impede treatment outcome.

Concerning inpatients, we have previously reported that at the end of treatment, highly dissociative patients, that is, diagnosed with a complex dissociative disorder (CDD; Dell, 2009), remained clinically worse-off than patients without these diagnoses, especially regarding dissociative symptoms and interpersonal functioning (Jepsen et al., 2013). The varied results among outpatients as well as the lack of studies based on inpatients point to the need for further research to clarify the relationship of severity of dissociation to treatment outcome among complex trauma inpatient populations, in order to better inform clinical practice in tailoring treatments to patient profiles.

Furthermore, difficulties in interpersonal functioning, such as mistrust, emotional liability, and relational instability in chronically traumatized individuals, may lead to increased reluctance to engage in treatment and decreased effectiveness of treatment (Davis & Petretic-Jackson, 2000; Herman, 1992). Poor treatment outcome of general psychiatric symptoms following an inpatient trauma-based program has been linked to the presence of insecure attachment, whereas positive treatment outcome has been associated with the presence of social support (Stalker, Gebotys, & Harper, 2005).

In our daily practice, patients are often placed on a pretreatment waiting list before coming into inpatient treatment. The patients usually stay in their local environment before being transferred to the hospital setting. During the waiting period, they may be in their home settings wherein they are experiencing progressively more stress and interpersonal difficulties. A decrease in interpersonal functioning during a pretreatment waiting period prior to admittance to inpatient setting may reflect difficulties in the patient's local environment, that is, a reaction to current stress and lack of skills to deal more adequately with stressors, a sign of the patient being destabilized. This may have important effects on the treatment process and outcome (Baars et al., 2011; Myrick, Brand, & Putnam, 2013). Our clinical impression from a specialized 3-month inpatient treatment program for adults with CSA-related disorders suggested that this might be the case. To our knowledge, this has not previously been studied. In addition, after the end of the inpatient stay, where they have lived in a different setting, most patients return back to their usual living environment. We do not know whether the patients who showed a decrease in interpersonal functioning during a pretreatment waiting period stay on a consistent global symptom severity level and we also do not know if they are relatively stable in terms of interpersonal functioning after returning to their normal lives following inpatient treatment, given the paucity of published data in this area.

In particular, we would expect the interaction of a worsening of interpersonal functioning prior to treatment and severe dissociative problems to negatively predict long-term outcome in patients following an inpatient program for adults with CSA histories and mixed trauma-related disorders, after they have returned to their local interpersonal environment, rather than dissociation in isolation or consistency in a low functioning level.

Information about the impact of initial pathological dissociation on treatment outcome is lacking, as well as information about the impact of a change in interpersonal functioning. The present study attempts to fill that gap in the literature by examining whether severe levels of pathological dissociation and a pretreatment negative change in interpersonal functioning are associated with posttreatment (immediate and at 1-year follow-up) levels of general psychopathology and interpersonal functioning. The study was based on a specialized 3-month inpatient treatment program for early sexually abused adults with mixed trauma-related disorders.

## Method

### Procedure and participants

The study is a naturalistic follow-up study with four assessment points: pre-care evaluation, admission, discharge, and 1-year follow-up. The mean duration time from pre-care assessment to admission was 11.2 months (*SD=*6.25; range 1.9–28.7) due to unavoidable variation in applications for treatment and hospital capacity. The drop-out rate from the pretreatment waiting list was very low (*n=*4, 3%).

The selection criteria for admission to the program were: at least 18 years old at admission, having a CSA history by a caretaker or a person in authority over them before the age of 16 years, and having a PTSD and/or other trauma-related disorders. Exclusion criteria included current psychosis, acute psychiatric and medical conditions requiring emergency hospitalization, and organic conditions interfering with dissociative symptoms.

The current study used data gathered in the Jepsen et al. (2013) study, which included a total of 56 patients (52 women and 4 men). Eight of these patients were omitted from the analysis because of missing data.

The remaining patients (45 women and 3 men) constituted the current study sample. Their mean age was 38.9 years (*SD*=8.16; range 25–58). Thirty-one patients (64.6%) were married or living with a partner. Patients’ CSA histories included accumulated childhood interpersonal trauma and adult revictimization. Diagnostic information of DSM-IV-TR (American Psychiatric Association, 2000) Axis I diagnoses was obtained by the administration of respectively the Mini International Neuropsychiatric Interview (MINI; Sheehan et al., 1998) at pre-care evaluation and the Structured Clinical Interview for DSM-IV Dissociative Disorders-Revised (SCID-D-R; Steinberg, Hall, Lareau, & Cicchetti, 2000) at the beginning of treatment.

Forty-three (89.5%) of the 48 patients were diagnosed with a PTSD and 22 patients (45.8%) had a DSM-IV dissociative identity disorder (DID; *n=*4, 8.3%) or dissociative disorder not otherwise specified, subtype 1 (DDNOS-1; *n=*18, 37.5%), hereafter referred to as complex dissociative disorders (CDD; Dell, 2009). According to Dell (2009), CDDs are disorders of pathological dissociation, involving memory and identity.

The PTSD patients had the following rates of comorbid disorders: affective (93.0%), anxiety (excl. PTSD; 86.0%), somatoform (67.4%), CDD (48.8%), eating (14.0%), and alcohol/drug dependency (4.7%). The remaining five patients (10.4%) without PTSD fulfilled DSM-IV criteria for affective (depressive) disorders (100.0%), anxiety disorders (80.0%), somatoform disorders (60.0%), eating disorders (20.0%), and CDD (20.0%).

The Regional Ethical Committee approved the study. All participants were informed about the study and agreed to participate.

### Treatment

The study was conducted at the Unit for Trauma Treatment at Modum Bad, a national psychiatric clinic in Norway. The unit offered a 3-month specialized inpatient trauma treatment program for adults with a history of CSA and mixed trauma-related disorders.

The program followed guidelines of first phase trauma treatment, that is, symptom reduction and development of stabilization skills (e.g., Herman, 1992). It included individual and group therapy, and involved psychodynamic, cognitive-behavioral, and supportive interventions. The relational context was emphasized: patients were encouraged to use the context of the inpatient setting to elicit change in maladaptive behavior in the present linked with past traumatic experiences, into more adaptive behavior, including relational work such as sound self-assertiveness and limit setting. Important relatives were admitted to the hospital for a 4-day weekend stay for education and to strengthen supportive relationships. For a more detailed description of the treatment program, see Jepsen et al. (2009).

### Instruments

General psychiatric symptoms were measured with the Symptom Check List 90 Revised (SCL-90-R), a psychometrically well-validated scale (Derogatis, Lipman, & Covi, 1973). We used the Global Severity Index (GSI) to measure general psychopathology. Higher values indicate greater distress. A cutoff of 0.85 on the GSI has been used to differentiate between normal and clinical levels of symptoms (Pedersen & Karterud, 2004). Cronbach's α ranged from 0.96 to 0.98 across three measure points (admission, discharge, follow-up).

The psychometrically sound Inventory of Interpersonal Problems (IIP-C, Norwegian version; Pedersen, 2002) was used to measure interpersonal problems. Higher values indicate greater problems, with a mean value above 1 indicating significant interpersonal problems. Cronbach's α ranged from 0.89 to 0.96 across the four measure points (pre-care evaluation, admission, discharge, follow-up). The change from pre-care evaluation to admission, defined as the difference (ΔIIP), was calculated for each patient and used as a predictor in the analysis. In two of the prediction models, IIP-C was also used as an outcome measure.

The 8-item taxonomic version of the Dissociative Experiences Scale (DES-T) was used to measure dissociative symptoms (Waller, Putnam, & Carlson, 1996). We used the cutoff score of 20+ on the DES-T (Waller & Ross, 1997) as a categorical index for identifying individuals with severe levels of pathological dissociation. Cronbach's α for the DES-T was 0.89 at admission and 0.93 at discharge.

The current study sample included 18 patients (37.5%) identified as DES-T members (i.e., with severe pathological dissociation) at admission and discharge (*r=*0.91, *p<*0.01). DES-T membership was significantly correlated with a CDD diagnosis at admission (*r=*0.76, *p<*0.01) and at discharge (*r=*0.84, *p<*0.01). At admission, 17 (94.4%) of 18 DES-T members had a CDD diagnosis (DDNOS-1: *n*=13, 72.2%; DID: *n*=4, 22.2%). At discharge, all patients with a DES-T membership had a CDD diagnosis (DDNOS-1: *n=*14, 77.8%; DID: *n*=4, 22.2%). For more details on the assessment of the dissociative disorders, see Jepsen et al. (2013).

### Analyses

The data were tested and found to satisfy the assumptions for parametric tests. Analysis of variance (ANOVA) with repeated measures was performed for the SCL-90-R GSI and the IIP-C to investigate outcome across time from admission and discharge to follow-up. Cohen's effect sizes within groups were reported as the standardized difference of means at each time point (Cohen, 1988). Associations were determined using Pearson's correlation. Hierarchical regression was used to determine if pathological dissociation (DES-T membership), the change from pre-care to admission in interpersonal problems (ΔIIP), as well as their interaction (DES-T × ΔIIP) were predictive of treatment response, while controlling for initial score on the outcome measure. Four separate hierarchical regression analyses were performed, one for the treatment period and one for the follow-up period for each of the outcome variables, the SCL-90-R GSI and IIP-C. In these analyses, the initial level of the outcome measure (i.e., the admission level for the treatment period and the discharge level for the follow-up period) was entered in the first step, and the three predictor variables in the second step. The DES-T membership at admission was used in the analyses for the treatment period, and the DES-T membership at discharge was used in the analyses for the follow-up period.

Four additional analyses were performed with presence of a CDD (yes/no) substituting the DES-T membership in the predictive models. This approach provided us with the opportunity to also assess whether it is relevant to consider a CDD in the context of inpatient treatment for polysymptomatology related to child abuse.

The significance level was set at *p*<0.05 (two-tailed). Data were analyzed using SPSS version for 19.0 Windows.

## Results

Table 1 shows the means and standard deviations of the study sample for the SCL-90-R GSI and the IIP-C at three and four measure points, respectively, as well as Pearson correlations between the dependent and independent variables. Because correlations among some of the predictor variables were high, we checked whether the assumption of multicollinearity was violated, which was not the case.

**Table 1:** Descriptive statistics and bivariate correlations for the main variables

Variable	M (SD)	1	2	3	4	5	6	7	8	9	10
1. Pre-care IIP	1.83 (0.40)	–									
2. Admission IIP	1.83 (0.38)	0.65**	–								
3. Discharge IIP	1.65 (0.45)	0.50**	0.57**	–							
4. Follow-up IIP	1.55 (0.59)	0.44**	0.51**	0.65**	–						
5. Admission SCL-GSI	1.86 (0.59)	0.52**	0.63**	0.53**	0.43**	–					
6. Discharge SCL-GSI	1.58 (0.74)	0.44**	0.40**	0.73**	0.55**	0.76**	–				
7. Follow-up SCL-GSI	1.46 (0.77)	0.33**	0.42**	0.45**	0.74**	0.60**	0.71**	–			
8. ΔIIP	0.01 (0.33)	−0.46**	0.38**	0.05	0.06	0.11	−0.06	0.10	–		
9. Admission DES-T		0.23	0.28	0.50**	0.27	0.53**	0.57**	0.34**	0.06	–	
10. Discharge DES-T		0.27	0.32*	0.55**	0.32*	0.53**	0.59**	0.39**	0.05	0.91**	–

Note: (N=48).

IIP=Inventory of Interpersonal Problems Circumplex; SCL-GSI=Symptom Check List Revised Global Severity Index; ΔIIP=difference between IIP at pre-care and admission. DES-T=DES-Taxon membership.

* Correlation is significant at the 0.05 level (2-tailed).

** Correlation is significant at the 0.01 level (2-tailed).

During the pretreatment waiting period (i.e., from pre-care evaluation to admission), there was no significant change in neither general psychiatric symptoms, *F*(1, 47)=0.061, *ns*, nor interpersonal problems, *F*(1, 47)=0.025, *ns*. Across time, from admission to follow-up, overall statistical significant improvement was found for general psychiatric symptoms, *F*(2, 94)=12.540, *p<*0.001 as well as for interpersonal problems, *F*(2, 94)=9.714, *p<*0.001. During the treatment period (admission to discharge), there were significant improvements on both measures. These gains were maintained with no further significant improvements in the 1-year follow-up period. Cohen's effect-sizes from admission to follow-up were moderate: 0.60 for both outcome measures. Changes in interpersonal problems and general psychiatric symptoms correlated significantly in the pretreatment period (*r=*0.50, *p<*0.01), during the inpatient period (*r=*0.69, *p<*0.01), as well as in the follow-up period (*r=*0.70, *p<*0.01).

The results of the regression analyses are shown in Table 2. While controlling for the admission score of SCL-90-R GSI, DES-T membership at admission was a significant predictor of greater general psychiatric symptoms at discharge (*F*(4,43)=18.97, *p<*0.001) (Table 2). A parallel model computed over the 1-year follow-up yielded a significant effect of the interaction term (Discharge DES-T × ΔIIP) on general psychiatric symptoms, while controlling for the discharge SCL-90-R GSI score (*F*(4,43)=13.93, *p<*0.001) (Table 2).

**Table 2:** Hierarchical regression predicting levels of general psychiatric symptoms and interpersonal problems at discharge (1 and 2) and 1-year follow-up (3 and 4)

Treatment period	*β*value	[95% CI]	*R* ^2^	*R* ^2^ Δ	Follow-up period	*β*value	[95% CI]	*R* ^2^	*R* ^2^Δ
*SCL-GSI at discharge (1)*					*SCL-GSI at follow-up (3)*				
Predictor variable					Predictor variable				
Step 1			0.58	0.58***	Step 1			0.50	0.50***
Admission SCL-GSI	0.76***	0.72–1.20			Discharge SCL-GSI	0.71***	0.52–0.96		
Step 2			0.64	0.06	Step 2			0.56	0.07
Admission SCL-GSI	0.65***	0.55–1.10			Discharge SCL-GSI	0.72***	0.48–1.02		
Admission DES-T	0.23*	0.01–0.67			Discharge DES-T	−0.04	−0.47–0.33		
ΔIIP	−0.16	−0.91–0.17			ΔIIP	−0.04	−0.72–0.54		
Admission DES-T × ΔIIP	0.03	−0.74–0.97			Discharge DES-T × ΔIIP	0.28*	0.07–2.04		
*IIP at discharge (2)*					*IIP at follow-up (4)*				
Predictor Variable					Predictor Variable				
Step 1			0.32	0.32***	Step 1			0.43	0.43***
Admission IIP	0.57***	0.38–0.95			Discharge IIP	0.65***	0.56–1.14		
Step 2			0.49	0.17**	Step 2			0.51	0.08
Admission IIP	0.54***	0.34–0.93			Discharge IIP	0.71***	0.59–1.26		
Admission DES-T	0.37**	0.13–0.55			Discharge DES-T	−0.08	−0.41–0.22		
ΔIIP	−0.06	−0.49–0.33			ΔIIP	−0.20	−0.87–0.14		
Admission DES-T × ΔIIP	−0.18	−1.02–0.22			Discharge DES-T × ΔIIP	0.36*	0.23–1.83		

*Notes:* (*N*=48). Results are given as regression coefficients (*β*values) and 95% confidence intervals [95% CI].

SCL-GSI=Symptom Check List Revised Global Severity Index; IIP=Inventory of Interpersonal Problems Circumplex; ΔIIP=difference between IIP at pre-care and admission; DES-T=DES-T membership.

* *p<*0.05;

** *p<*0.01;

*** *p<*0.001.

A similar pattern of findings was found for the two models computed over the social/interpersonal outcomes: While controlling for the admission score of IIP-C, DES-T membership at admission significantly predicted interpersonal outcome at discharge (*F*(4,43)=10.33, *p<*0.001) (Table 2). Additionally, the interaction term (Discharge DES-T × ΔIIP) was a significant predictor of greater interpersonal problems at follow-up while controlling for the discharge IIP-C score (*F*(4,43)=11.03, *p<*0.001) (Table 2).

When substituting DES-T membership with CDD as the predictor variable in the regression model, we obtained similar results (data not shown).

## Discussion

Our hypothesis that pathological dissociation and increased interpersonal problems during a pretreatment waiting period would predict less favorable outcome following a specialized 3-month inpatient program for complex trauma patients with different trauma-related disorders was supported by the findings. More specifically, pathological dissociation in isolation significantly predicted higher levels of general psychiatric symptoms and lower interpersonal functioning at discharge, but not at 1-year follow-up. The interaction between pathological dissociation at discharge and pretreatment change in interpersonal functioning significantly predicted outcome at 1-year follow-up.

Our findings that pathological dissociation was related to negative outcome in terms of higher levels of general psychiatric symptoms differ from the findings reported in the outpatient studies by Lynch et al. (2008) and Brand and Stadnik (2013). A possible explanation for the diversities might be differences in study designs (e.g., inpatients vs. outpatients, pathological dissociation vs. dissociation, focus of treatment) or differences in study populations (e.g., in psychopathology, number of times in prior treatment, remaining in treatment). Nine percent of the sample of Lynch et al. (2008) was diagnosed with a dissociative disorder compared to 45.8% in the current study. All patients (100%) in the Brand and Stadnik study (2013) were in treatment for a severe dissociative disorder and may have received treatment that partially differed in treatment focus compared to the current program.

Our results indicate that the stabilizing inpatient treatment program for adults with histories of CSA and different trauma-related disorders was more beneficial for patients without pathological dissociation than for patients with this condition. The program did not specifically target pathological dissociation involving memory and identity, which may have contributed to the persistence of greater global symptom severity distress in patients with these problems. Overall, our findings confirm the clinical utility of identifying traumatized patients with PTSD and severe levels of dissociation, and tailoring treatment to this patient group (Cloitre et al., 2012; Lanius et al., 2010; Resick et al., 2012). Although the use of the DES-T for identification of patients with severe dissociative disorders has been questioned (e.g., Modestin & Erni, 2004), our data support the utility of the DES-T for a preliminary identification of patients with severe dissociative problems in a complex trauma inpatient population. Being a clinician-friendly instrument, this could facilitate the identification of patients with severe dissociative problems when a full diagnostic assessment for dissociative disorders is not feasible. Furthermore, our findings at 1-year follow-up indicate that it is not pathological dissociation in isolation that affects outcomes in our inpatient sample, but rather the interaction between dissociation and negative change in interpersonal functioning prior to treatment. These findings indicating that severe dissociation combined with deterioration in interpersonal functioning in the home setting prior to the inpatient stay contributed to greater general psychopathology and lower levels of interpersonal functioning after they had returned to their usual lives, suggest that contextual factors influence these forms of distress and functioning. However, the underlying causes for exacerbation of problems in interpersonal relations distress prior to hospital admittance and following treatment could not be identified.

Some possible factors that may be relevant include the patient's relational environment (family, partner, social network, work) (e.g., Benjamin & Benjamin, 1994; Sachs, Frischholz, & Wood, 1988) and life-stressors (traumatic or non-traumatic) in the patient's home setting, as for example, revictimization, marital problems, housing changes, lack of social support, lack of resources (Myrick et al., 2013). In a qualitative study of 30 patients’ feedback on a trauma-based inpatient program for adults with childhood abuse histories, many patients reported that they “returned to an unchanged world,” with the only difference being that they now recognized the difficulties at home, including dysfunctional relationships (Palmer, Stalker, Gadbois, & Harper, 2004).

Therefore, ongoing life-stressors in the patient's home setting, combined with increased realization of difficulties in interpersonal functioning and lack of sufficient skills to deal with the situation, may have contributed to increased self-reports of general psychopathology scores at follow-up in the most troubled patients in our sample. Although our treatment program involved individual and group therapy as well as a 4-day weekend stay for important relatives, this may not have been sufficient to achieve lasting gains in the sense of relatively consistent global symptom severity as well as interpersonal functioning after returning to their normal lives in severely dissociative patients.

Although our study improves on prior research in several ways, it has a number of limitations, so the findings should be viewed with caution. First, our sample was small, reducing power to find significant differences. Secondly, the study sample consisted of adult patients with CSA histories in need of an inpatient treatment program, so the findings cannot be extended to general populations or other patient groups. Future research should also include outcome data not based on patients’ self-report. Finally, attachment and Axis II disorders as well as medication were not examined; all of these might have affected treatment response.

## Clinical implications

Our results suggest the clinical utility of identifying patients with pathological dissociation or CDDs. The DES-T may be useful for this purpose as a first step in the process of determining appropriate treatment. Besides profiling based on the severity of dissociative symptoms, it also seems important to consider interpersonal functioning, especially any exacerbation of interpersonal problems, in determining treatment components. The underlying causes for a pretreatment deterioration in functioning (e.g., non-traumatic as well as traumatic life-stressors in their local environment) should be identified.

The inclusion of the 1-year follow-up provides practical evidence for clinicians working with members of complex traumatized populations. Study findings indicating that the interaction between interpersonal functioning change and dissociation predict outcome at 1-year follow-up particularly favor an integrative treatment approach. The optimal treatment strategy for these highly dissociative patients might be phase-based treatment that specifically addresses the dissociative problems (International Society for the Study of Trauma and Dissociation, 2011) in addition to the general sequenced approach recommended for treatment of chronic traumatization (Courtois & Ford, 2013; Herman, 1992). As suggested by treatment guidelines (ISSTD, 2011), severely dissociative patients in first-phase treatment should identify and modify disordered attachment patterns learned in childhood, and work on competence in social interactions in parallel with development of affect regulation and grounding skills. Manualized stabilizing group treatment, including building interpersonal and affect regulation skills and specific address of dissociative problems, might be added to the program (e.g., Boon, Steele, & Van der Hart, 2011; Cloitre, Cohen, & Koenen, 2006; Dorrepaal et al., 2012). Strengthening of contextual approaches might be considered in severely dissociated patients who deteriorated in their home setting prior to treatment (e.g., Gold & Seibel, 2009).

## Conclusion

Our findings that severe forms of dissociation involving memory and identity were associated with poorer outcome following an inpatient treatment program, support the importance of addressing these problems in treatment planning for complex trauma populations. Furthermore, our findings indicate the importance of paying attention to patients’ pretreatment functioning and possible underlying causes for any deterioration in interpersonal functioning, in particular for patients with severe dissociative problems. Overall, our findings support the philosophy of building interpersonal skills in parallel with stabilization of pathological dissociation in the treatment of highly dissociative patients. Future research should investigate if this leads to better outcome, including long-term maintenance of gains after the end of treatment.
